# Synthesis, reactions and biological activity of some new bis-heterocyclic ring compounds containing sulphur atom

**DOI:** 10.1186/1752-153X-7-112

**Published:** 2013-07-08

**Authors:** Yahia Nasser Mabkhot, Assem Barakat, Abdullah Mohammed Al-Majid, Saeed Alshahrani, Sammer Yousuf, M Iqbal Choudhary

**Affiliations:** 1 Department of Chemistry, Faculty of Science, King Saud University, P. O. Box 2455, Riyadh 11451, Saudi Arabia; 2 Department of Chemistry, Faculty of Science, Alexandria University, P.O. Box 426, Ibrahimia 21321, Alexandria, Egypt; 3 H.E.J. Research Institute of Chemistry, International Center for Chemical Sciences, University of Karachi, Karachi 75270, Pakistan

**Keywords:** Thienothiophene, Oxazole, Imidazole, Thiazole, *Bis*heterocycles, *β*-glucuronidase inhibition, *α*-glucosidase inhibition, DPPH radical scavenging activity, Ctotoxicity, Cancer cell line

## Abstract

**Background:**

The derivatives of thieno[2,3-*b*]thiophene belong to a significant category of heterocyclic compounds, which have shown a wide spectrum of medical and industrial application.

**Results:**

A new building block with two electrophilic center of thieno[2,3-*b*]thiophene derivatives **2** has been reported by one-pot reaction of diketone derivative **1** with Br_2_/AcOH in excellent yield. A variety of heteroaromatics having bis(1H-imidazo[1,2*a*] benzimidazole), bis(1H-imidazo[1,2-*b*][1,2,4]triazole)-3-methyl-4-phenylthieno[2,3-*b*]thiophene derivatives, dioxazolo-, dithiazolo-, and 1H-imidazolo-3-methyl-4-phenylthieno[2,3-*b*]thiophene derivatives as well pyrrolo, thiazolo -3-methyl-4-phenylthieno[2,3-*b*]thiophene derivatives have been designed, synthesized, characterized, and evaluated for their biological activity. Compounds **3**–**9** showed good bioassay result. These new derivatives were evaluated for anti-cancer activity against PC-3 cell lines, *in vitro* antioxidant potential and *β*-glucuronidase and *α*-glucosidase inhibitory activities. Compound **3** (IC_50_ = 56.26 ± 3.18 *μ*M) showed a potent DPPH radical scavenging antioxidant activity and found to be more active than standard *N*-acetylcystein (IC_50_ = 105.9 ± 1.1 *μ*M). Compounds **8a** (IC_50_ = 13.2 ± 0.34 *μ*M) and **8b** (IC_50_ = 14.1 ± 0.28 *μ*M) found as potent inhibitor of *α*-glucusidase several fold more active than the standard acarbose (IC_50_ = 841 ± 1.73 *μ*M). Most promising results were obtained in *β*-glucuronidase enzyme inhibition assay. Compounds **5** (IC_50_ = 0.13 ± 0.019 *μ*M), **6** (IC_50_ = 19.9 ± 0.285 *μ*M), **8a** (IC_50_ = 1.2 ± 0.0785 *μ*M) and **9** (IC_50_ = 0.003 ± 0.09 *μ*M) showed a potent inhibition of *β*-glucuronidase. Compound **9** was found to be several hundred fold more active than standard D-Saccharic acid 1,4-lactone (IC_50_ = 45.75 ± 2.16 *μ*M).

**Conclusions:**

Synthesis, characterization, and in vitro biological activity of a series of thieno[2,3-*b*]thiophene have been investigated.

## Background

Thieno[2,3-*b*]thiophenes represent a class of heterocyclic compounds endowed with potent antitumor and antiviral activity,[1-7] In particular, thienothiophene derivatives are reported as antiglaucoma drugs, as inhibitors of platelet aggregation, or as antibitotic [8-12]. Annulation of heterocyclic moieties on the thieno[2,3-*b*]thiophene nucleus led to the formation of diverse hetero analogues, which exhibited remarkable chemical and biological activities. For the past few years, Various protocols have been prepared and evaluated biologically important compounds derived from thieno [2,3-*b*]thiophene [13-28]. We have reported for the first time the anti-cancer, anti-oxidant and *β*-glucuronidase and *α*-glucosidase inhibition potential of thieno[2,3-*b*]thiophenes based molecules [20]. Furthermore, Studies revealed that compounds with nitrogen-oxygen- and sulfur containing heterocycles are chemotherapeutics available. Thiazoles and their derivatives have attracted much attention due to their wide range of biological and pharmacological activities, Such as treat allergies, [29] schizophrenia, hypertension, inflammation, bacterial and HIV infections [30-35].

The promising results of previous studies [20,36-38] prompted us to further extend our research towards the synthesis of annulation of heterocyclic systems of potential biological application. In continuation of our previous work we are reporting here the synthesis of some more analogues of thieno[2,3-*b*]thiophene moiety as a base unit and their *in vitro* anti-oxidant activity, including *α*-glucosidase and *β*-glucuronidase inhibition and anticancer activity against PC-3 cell lines.

## Results and discussion

### Chemistry

**Scheme 1:**
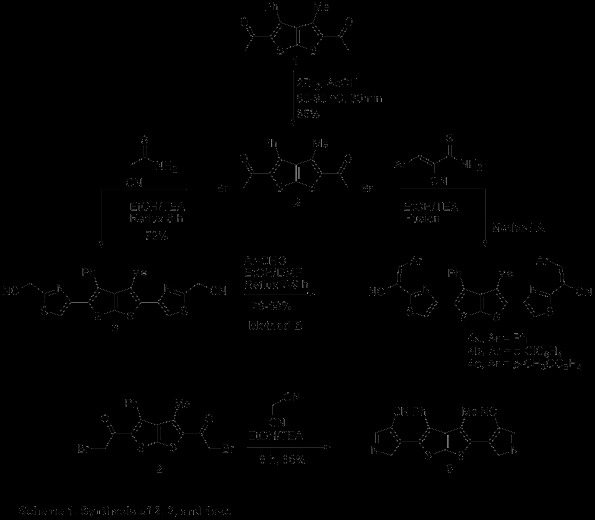
Synthesis of 2, 3, and 4a-c.

One possible synthetic strategy for the target bis(1H-imidazo[1,2*a*] benzimidazole) and bis(1H-imidazo[1,2-*b*][1,2,4]triazole)-3-methyl-4-phenylthieno[2,3-*b*]thiophene derivatives, dioxazolo-, dithiazolo-, and 1H-imidazolo-3-methyl-4-phenylthieno[2,3-*b*]thiophene derivatives as well pyrrolo, thiazolo-3-methyl-4-phenylthieno[2,3-*b*]thiophene derivatives as could have made use of bromoketone **2** (Scheme 1) as template for the annulation of the five member ring. Such intermediates were obtained from ketone of type **1**, about which not much is reported in literature.

Having in hand a new building block of starting ketones of type **1**, the next step would be the functionalization of a position to the carbonyl to introduce in the molecule a second electrophilic center that, together with the carbonyl group, should allow the cyclization with dinucleophiles. Direct introduction of the bromine functionality was also studied.

Thieno[2,3-*b*]thiophene derivatives **1** was converted into the corresponding bromoketone**2** (85%) using Br_2_ in refluxing AcOH for 1 h. The desired product was obtained by filtration, washed with water, dried well and recrystallized from ethanol to give white crystals.

To synthesize the bis heterocyclic system, we could react bromoketone **2** with 1,3-dinucleophiles having a C-C-N structure, such as cyanothioacetamide, 2-cyano-2-arylmethylene-thioacetamide, and malononitrile.

Compound **3** was synthesized by reaction of bromoketone **2** with suitably cyanothioacetamide under conventional reflux conditions in the presence of a catalytic amount of TEA using ethanol as a solvent. The structure of the isolated cycloadduct was determined by IR, ^1^H NMR, ^13^C NMR, mass spectral and elemental analyses. The utility of **3** towards suitably aldehydes for example benzaldehyde, *p*-chlorobenzaldehyde, *p*-methoxybenzaldehyde was also investigated. Compounds **4a**-**c** were prepared by reaction of **3** with suitable aromatic aldehyde under conventional reflux conditions in the presence of a catalytic amount of TEA using ethanol as a solvent. Alternatively, compounds **4a**-**c** were obtained by the fusion of thieno[2,3-*b*]thiophene derivative **2** with 2-cyano-2-arylmethylene-thioacetamide neat (Scheme 1). On the other hand, compound **5** was synthesized by treating the corresponding bromoketone **2** with malononitrile by thermal intramolecular cyclization reaction *via* an initial Michael type adduct. The IR(KBr) spectrum of compound **5**, exhibit absorption band due to the stretching vibrations of CN group at 2212 cm^-1^. The later compound was also confirmed by ^1^H-NMR spectrum exhibited signals at *δ* 1.20, 1.90, and 3.90, due to CH_3_, CH_2_, and CH pyrrole protons respectively, in addition to an aromatic multiplet in the region of δ 7.53–7.57. Its mass spectrum showed the molecular ion peak at *m/z* 410 (see Additional file 1).

**Scheme 2:**
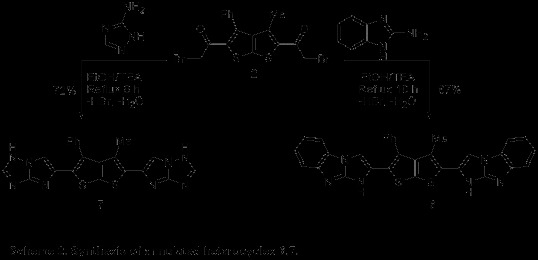
Synthesis of annulated heterocycles 6,7.

Annulated heterocycles was further developed *via* reaction of bromoketone**2** with different nucleophiles likes 2-aminobenzimidazole, 4-amino-1,2,4-triazole with a view to synthesizing various heterocyclic ring systems. Compounds **6**–**7** were synthesized by reaction of **2** with suitably amine derivatives under conventional reflux conditions in the presence of a catalytic amount of TEA using ethanol as a solvent affording the desired product **6** (87%) and **7** (72%)(Scheme 2). The ^1^H-NMR (DMSO-*d*_6_) spectrum of the compound **6** revealed three singlets signal at *δ* 1.96, 8.86, and 12.82 assigned to CH_3_, CH (imidazo-H), and NH (hydrogen-bonded with S) respectively. Its mass spectrum revealed a molecular ion peak at *m/z* 540. It is assumed that the product **7** was formed *via* initial formation of a nonisolable hydrazonal followed by elimination of H_2_O and HBr to give the desired product.

**Scheme 3:**
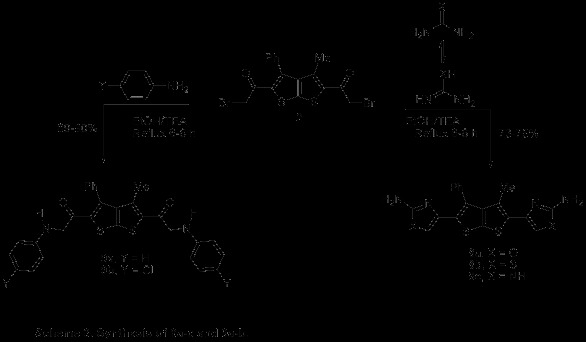
Synthesis of 8a–c and 9a-b.

Addition experiments of bromoketone **2** were carried out to afford the oxazole, thiazole, and 1H-imidazole after an elimination/aromatization of the cycloadduct intermediate. Conventional heating of bromoketone **2** with the corresponding *N*-nucleophile urea derivatives derivative at reflux temperatures had to be employed for the synthesis of oxazole, thiazole, and 1H-imidazole derivatives **8a**-**c** were synthesized following the conventional procedure bromoketone **2** with in ethanol at reflux in very good yield as depicted in (Scheme 3). Compounds **8a**-**c** were supposed to be formed *via* stepwise formation of hydrazone followed by a Michael 1,4-addition of the nucleophile nitrogen atom.

The structure of the desired compound **8a** was deduced by the ^1^H-NMR (DMSO-*d*_6_) spectrum which displayed a three singlet’s signal at *δ* 1.79, 6.76, and 7.54 assignable to CH_3_, NH_2_, and CH of oxazole ring respectively. The formation of compound **8a** would involve an initial addition of the amino group in urea to the electrophilic center of bromo functionalities in bromoketone **2**, then elimination of HBr subsequently cyclization and aromatization *via* loss of water gave the final desired compound (Scheme 3). The later hypothesis has been confirmed by reaction only bromoketone **2** (dielectrophilic centers) with only one nucleophilic center such as amine derivatives. Thus refluxing bromoketone **2** with aniline derivatives in EtOH for 6-8 h affording **9a**-**b** in excellent yield (Scheme 3). Spectral data (IR, NMR, MS) and elemental analysis were consistent with isolated product **9a**. The ^1^H-NMR spectrum of **9a** showed three singlets signal at *δ* 2.02, 4.42 and 7.61 due to CH_3_, CH_2_, and NH protons, in addition IR spectrum revealed absorption band at 1653, and 3385 cm^-1^ corresponding to two C = O and amino functions, respectively. Its mass spectrum revealed a molecular ion peak at *m*/*z* 496 it means that doesn’t contain a Br atom.

### Biological activity evaluation

Compounds **3**–**9** were evaluated for potential biological activities through a battery of *in vitro* biochemical assays including anticancer activity against PC-3 cell lines, antioxidant potential in DPPH radical scavenging assay and *β*-glucuronidase and *α*-glucosidase enzyme inhibition assays. The results are presented in Table 1.

**Table 1:** **Results of various biological assays on compounds 3**–**9**

**Compounds**		**IC**_**50**_ ± **SEM** [μ**M**]		
	**Anticancer activity** (**PC**-**3 cell line**)	**DPPH radical scavenging assay**	***ß***-**Glucuronidase inhibition**	***α***-**Glucosidase inhibition**
**3**	>30	**56**.**26** ± **3**.**18**	NA	NA
**4a**	>30	NA	-	-
**4b**	>30	NA	NA	NA
**5a**	>30	NA	**0**.**130** ± **0**.**019**	-
**5b**	>30	NA	**19**.**9** ± **0**.**285**	-
**6**	>30	NA	**1**.**2** ± **0**.**0785**	-
**7**	>30	NA	NA	-
**8a**	>30	-	**1**.**2** ± **0**.**0785**	**13**.**2** ± **0**.**34**
**8b**	**24**.**213** ± **0**.**29**	-	NA	**14**.**1** ± **0**.**28**
**9a**	>30	NA	**0**.**003** ± **0**.**09**	NA
**9b**	>30	NA	NA	-
**Std**.	**Doxorubicin 0**.**91** ± **0**.**1**	**N**-**Acetylcysteine 106** ± **1**.**1**	**D**-**Saccharic acid 1**,**4**- **lactone 45**.**75** ± **2**.**16**	**Acarbose 841** ± **1**.**7**

SEM = standard error of mean.

NA = not active; failed to show 50% or more inhibition at 500 μM or more.

Compound **3** (IC_50_ = 1.3 ± 0.172 *μ*M) showed a potent antioxidant potential in DPPH radical scavenging assay and found to be more active than the standard *N*-acetylcystein (IC_50_ = 105.9 ± 1.1 *μ*M). All other compounds found to be inactive. Compounds **5** (IC_50_ = 0.13 ± 0.019 *μ*M), **6** (IC_50_ = 19.9 ± 0.285 *μ*M), **8a** (IC_50_ = 1.2 ± 0.0785 *μ*M) and **9** (IC_50_ = 0.003 ± 0.09 *μ*M) showed promising results for *β*-glucuronidase inhibition activity and found to be several fold more active than the standard D-Saccharic acid 1,4-lactone (IC_50_ = 45.75 ± 2.16 *μ*M). 2-Aminoxazole and 2-aminothiazole substituted *bis-*thiazole ring containing compounds **8a** (IC_50_ = 13.2 ± 0.34 *μ*M) and **8b** (IC_50_ = 14.1 ± 0.28 *μ*M) showed potent *α*-glucosidase enzyme inhibition. Compound **8b** also found as moderate anticancer agent (IC_50_ = 24.213 ± 0.29 *μ*M) against PC-3 cell lines while tested against standard drug doxorubicin (IC_50_ = 0.912 ± 0.12 *μ*M) as. All other compounds (**3**-**8a**, **9**) were found to be non cytotoxic and showed >30% inhibition of PC-3 cancer cell lines.

## Conclusions

In conclusion, we have successfully developed an easy practical access to novel and readily accessible building block **2** for the synthesis of biologically important compounds incorporating thieno[2,3-*b*]thiophene core (**3**–**9**). These compounds were evaluated for their biological activities in various *in vitro* biological assays. The potent antioxidant and *α*-glucosidase inhibiting activities of compounds **3** and **8a**, respectively, indicates their potential as possible leads for the treatment of oxidative stress and hyperglycemia associated health disorders. The most promising results of *β* -glucuronidase enzyme inhibitors **5**, **6**, **8a** and **9** can serve as templates for the new drug candidates for the treatment of cancer, rheumatoid arthritis, AIDS and other health problems associated with over expression of *β* -glucuronidase enzyme.

## Experimental section

### General

All melting points were measured on a Gallenkamp melting point apparatus. IR spectra were measured as KBr pellets on a Perking Elmer FT 1000 spectrophotometer. The NMR spectra were recorded on a Varian Mercury Jeol-400 NMR spectrometer. ^**1**^H-NMR (400 MHz) and ^**13**^C-NMR were run in dimethylsulphoxide (DMSO-*d*_6_). Chemical shifts (δ) are referred in terms of ppm and J -coupling constants are given in Hz. Abbreviations for multiplicity is as follows: s (singulet), d (doublet), t (triplet), q (quadruplet), m (multiplet). Mass spectra were recorded on a Shimadzu GCMS-QP 1000 EX mass spectrometer at 70 eV. Elemental analysis was carried out on an Elementar Vario EL analyzer.

### 1,1'-(3-Methyl-4-phenylthieno[2,3-b]thiophene-2,5-diyl)bis(2-bromoethanone) (2)

A mixture of **1** (3.14 g, 10 mmol) in glacial acetic acid (100 mL). The reaction mixture was heated up to 80–90°C with vigorous stirring. To this hot solution, bromine (1.1 mL) in glacial acetic acid (20 mL) was added drop wise over a period of 30 min. After complete addition of bromine, the reaction mixture was stirred vigorously at room temperature for further 1 h till the release of hydrogen bromide gas ceased, then poured onto ice. The solid product was collected by filtration, washed with water, dried well and recrystallized from ethanol to give white crystals of **2**; Yield: 85%; solid, mp 142–144°C;IR (KBr) ν_max_/ cm^-1^: 1653; ^1^H-NMR (400 MHz, DMSO-*d*_6_)*δ* 1.95 (s, 3H, CH_3_) 4.73 (s, 4H, 2CH_2_), 7.52-7.58 (m, 5H, Ar-H); ^13^C-NMR (100 MHz, DMSO-*d*_6_)*δ* 185.2, 160.8, 149.7, 141.2, 137.9, 135.9, 133.3, 129.2, 128.5, 125.5, 35.5, 13.8; MS *m/z*(%): 472 [M^+^, 35%]; Anal. calcd. for C_17_H_12_Br_2_O_2_S_2_ : C, 43.24; H, 2.56; S, 13.58; Found:C,43.19; H, 2.58; S,13.21.

### 2,2'-(4,4'-(3-Methyl-4-phenylthieno[2,3-b]thiophene-2,5-diyl)bis(thiazole-4,2-diyl)) diacetonitrile (3)

A mixture of compound **2** (472 mg, 1 mmol) and 2-cyanoethanethioamide (200 mg, 2 mmol) was heated under reflux for 8 h in EtOH (15 mL), in the presence of 0.5 mL of (TEA). The solid product was collected by filtration to give dark brown powder crystals; Yield (72%); solid, mp > 320°C; IR (KBr) ν_max_/ cm^-1^: 2247 ; ^1^H-NMR (400 MHz, DMSO-*d*_6_)*δ* 1.84 (s, 3H, CH_3_), 3.45 (s, 4H, 2CH_2_), 6.3 (s, 2H, 2CH), 7.43-7.70 (m, 5H, Ar-H); ^13^C-NMR (100 MHz, DMSO-*d*_6_)*δ* 158.5, 158.2, 147.9, 147.7, 146.7, 135.0, 129.3, 128.9, 128.3, 127.3, 116.6, 115.8, 21.2, 13.4; MS *m/z*(%): 474[M+, 4%]; Anal. calcd. for C_23_H_14_N_4_S_4_: C, 58.20; H, 2.97; N, 11.80; S, 27.02; Found: C, 59.10; H, 2.86; N, 11.91; S, 26.12.

### Compounds 4a-c was prepared in two methods

#### *Method A (GP1)*

Fusion of compound **2** (236 mg, 0.5 mmol) with 2-cyano-3-arylprop-2-enethioamide derivatives (2 equiv., 1 mmol). The solid product was collected by filtration and washed with EtOH, dried and the crude product was recrystallized from EtOH / DMF to give the corresponding compounds (**4a**-**c**).

#### *Method A (GP2)*

A mixture of compound **3** (237 mg, 0.5 mmol) and aromatic aldehydes (2 equiv., 1 mmol) was refluxed in EtOH (15 mL) for 7–9 h in the presence of 0.5 mL of (DMF). The solid product was collected by filtration to give the corresponding products **4a**-**c**.

### 2-(4-(5-(2-(1-Cyano-2-phenylvinyl)thiazol-4-yl)-3-methyl-4-phenylthieno[2,3-b]thiophen-2-yl)thiazol-2-yl)-3-phenylacrylonitrile (4a)

**4a** was prepared from 2-cyano-3-phenylprop-2-enethioamide following GP1, and from benzaldehyde following GP2, as a pale brown powder crystals; Yield (88%^GP1^,79% ^GP2^); solid, mp 220–221°C; IR (KBr) ν_max_/ cm^-1^: 1606, 2212; ^1^H-NMR (400 MHz, DMSO-*d*_6_)*δ* 1.93 (s, 3H, CH_3_), 7.49-8.03 (m, 15H, Ar-H) 8.21 (s, 2H, 2CH), 8.30 (s, 2H, 2Ar-CH); ^13^C-NMR (100 MHz, DMSO-*d*_6_)*δ* 162.8, 146.1, 140.3,137.7, 136.4, 135.4, 133.5, 132.8, 130.2, 129.9, 129.1, 128.6, 125.4, 116.5, 115.8, 113.0, 14.3: MS *m/z*(%): 650[M+, 1.5%]; Anal. calcd. for C_37_H_22_N_4_S_4_: C, 68.28; H, 3.41; N, 8.61; S, 19.71; Found: C, 67.88; H, 3.30; N, 8.71; S, 19.41.

### 3-(4-Chlorophenyl)-2-(4-(5-(2-(2-(4-chlorophenyl)-1-cyanovinyl)thiazol-4-yl)-3-methyl-4-phenylthieno[2,3-b]thiophen-2-yl)thiazol-2-yl)acrylonitrile (4b)

**4b** was prepared from 2-cyano-3-(4-chlorophenyl)prop-2-enethioamide following GP1, and from 4-chlorobenzaldehyde following GP2, as a pale brown powder crystals; Yield (84%^GP1^ , 80% ^GP2^); solid, mp 167–168°C;IR (KBr) ν_max_/ cm^-1^: 1606, 2218; ^1^H-NMR (400 MHz, DMSO-*d*_6_)*δ* 1.97 (s, 3H, CH_3_), 7.48-8.05 (m, 15H, Ar-H) 8.16 (s, 2H, 2CH), 8.28 (s, 2H, 2Ar-CH); ^13^C-NMR (100 MHz, DMSO-*d*_6_)*δ* 162.8, 146.1, 139.8,137.2, 136.1, 135.1, 133.5, 130.2, 129.9, 129.1, 128.6, 127.2, 116.5, 115.8, 112.0, 14.3; MS *m/z*(%): 719[M+, 1.5%]; Anal. calcd. for C_37_H_20_Cl_2_N_4_S_4_: C, 61.74; H, 2.80; N, 7.78; S, 17.82; Found: C, 61.93; H, 2.76; N, 7.65; S, 17.49.

### 2-(4-(5-(2-(1-Cyano-2-(4-methoxyphenyl)vinyl)thiazol-4-yl)-3-methyl-4-phenyl thieno[2,3-b]thiophen-2-yl)thiazol-2-yl)-3-(4-methoxyphenyl)acrylonitrile (4c)

**4c** was prepared from 2-cyano-3-(4-methoxyphenyl)prop-2-enethioamide following GP1, and from 4-methoxybenzaldehyde following GP2, as a pale brown powder crystals; Yield (83%^GP1^, 80% ^GP2^); solid, mp 238–239°C; IR (KBr) ν_max_/ cm^-1^: 1606, 2212;^1^H-NMR (400 MHz, DMSO-*d*_6_)*δ* 1.96 (s, 3H, CH_3_), 3.85 (s, 3H, O-CH_3_), 7.14-8.01 (m, 15H, Ar-H), 8.23 (s, 2H, 2CH), 8.31 (s, 2H, 2Ar-CH); ^13^C-NMR (100 MHz, DMSO-*d*_6_)*δ* 162.8, 146.1, 140.3,138.9, 137.0, 135.9, 134.8, 132.3, 130.2, 129.9, 129.1, 128.6, 116.5, 115.7, 55.8, 14.3; MS *m/z*(%): 710[M+, 1.5%]; Anal. calcd. for C_39_H_26_N_4_O_2_S_4_: C, 65.89; H, 3.69; N, 7.88; S, 18.04; Found: C, 66.76; H, 3.59; N, 7.97; S, 18.74.

### 3-(5-(4-Cyano-2H-pyrrol-3-yl)-3-methyl-4-phenylthieno[2,3-b]thiophen-2-yl)-2H-pyrrole-4-carbonitrile (5)

A mixture of compound **2** (472 mg, 1 mmol) and malononitrile (132 mg, 2 mmol) was heated under reflux for 8 h in EtOH (15 mL), in the presence of 0.5 mL of (TEA). The solid product was collected by filtration to give dark purple powder crystals; Yield (66%); solid, mp > 320°C; IR (KBr) ν_max_/ cm^-1^: 2212;^1^H-NMR (400 MHz, DMSO-*d*_6_) *δ* 1.2 (s, 4H, 2CH_2_), 1.9 (s, 3H, CH_3_), 3.9 (s, 2H, 2CH), 7.53-7.57 (m, 5H, Ar-H); ^13^C-NMR (100 MHz, DMSO-*d*_6_)*δ* 159.6, 142.8, 138.7, 137.6, 135.0, 129.7, 129.3, 128.9, 128.3, 127.3, 115.8, 94.2, 55.9,13.4; MS *m/z*(%): 410[M+, 36%]; Anal. calcd. for C_23_H_14_N_4_S_2_: C, 67.29; H, 3.44; N, 13.65; S, 15.62; Found: C, 66.79; H, 3.49; N, 13.45; S, 15.78.

### 5,5'-(3-Methyl-4-phenylthieno[2,3-b]thiophene-2,5-diyl)bis(1H-imidazo[1,2a] benzimidazole) (6)

A mixture of compound **2** (236 mg, 0.5 mmol) and 2-aminobenzimidazole (133 mg, 1 mmol) was refluxed in EtOH (15 mL) for 10 h in the presence of 0.5 mL of triethyl amine (TEA). The resulting solid product was collected by filtration to give a reddish brown crystals; Yield (87%); solid, mp > 320°C; IR (KBr) ν_max_/ cm^-1^: 3373;^1^H-NMR (400 MHz, DMSO-*d*_6_)*δ* 1.96 (s, 3H, CH_3_), 7.37-7.25 (m, 13H, Ar-H), 8.86 (s, 2H, 2CH imidazo-H), 12.82 (s, 2H, 2NH); ^13^C-NMR (100 MHz, DMSO-*d*_6_)*δ* 157.2, 148.2, 147.6, 141.4, 138.5, 134.2, 129.2, 128.8, 125.0, 124.9, 124.5, 112.5, 1007.1, 15.8; MS *m/z*(%): 540[M+, 57%]; Anal. calcd. for C_31_H_20_N_6_S_2_: C, 68.87; H, 3.73; N, 15.54; S, 11.86; Found: C, 68.79; H, 3.76; N, 15.53; S, 11.89.

### 5,5'-(3-Methyl-4-phenylthieno[2,3-b]thiophene-2,5-diyl)bis(1H-imidazo[1,2-b] [1,2,4]triazole) (7)

A mixture of compound **2** (236 mg, 0.5 mmol) and 3-amino-1H-1,2,4-triazole (84 mg, 1 mmol) was heated under reflux for 8 h in EtOH (10 mL) in the presence of 0.5 mL of (TEA). The solid product was collected by filtration to give brown crystals; Yield (79%); solid, mp > 320°C; IR (KBr) ν_max_/ cm^-1^: 3410;^1^H-NMR (400 MHz, DMSO-*d*_6_)*δ* 1.92 (s, 3H, CH_3_), 7.52-7.42 (m, 5H, Ar-H), 8.52 (s, 2H, 2CH), 9.86 (s, 2H, 2 N = CH), 12.41 (s, 1H, NH); ^13^C-NMR (100 MHz, DMSO-*d*_6_) *δ* 163.0, 156.5, 148.4,144.4, 140.9, 137.8, 129.7, 124.0, 120.1, 15.4;MS *m*/*z*(%): 442[M+, 46%]; Anal. calcd. for C_21_H_14_N_8_S_2_: C, 57.00; H, 3.19; N, 25.32; S, 14.49; Found: C, 56.91; H, 3.22; N, 25.12; S, 14.59.

### General procedure for the synthesis of compounds 8a-c (GP3)

A mixture of compound **2** (0.472 g, 1 mmol), and urea derivatives (2 equiv., 2 mmol) was refluxed in EtOH (15 mL) for 6–8 h in the presence of 0.5 mL of (TEA). The solid product was collected by filtration to give the corresponding products **8a**-**c**.

### 4,4'-(3-Methyl-4-phenylthieno[2,3-b]thiophene-2,5-diyl)dioxazol-2-amine (8a)

**8a** was prepared from urea following GP3 as a brown powder crystals; Yield (73%); solid, mp > 320°C;IR (KBr) ν_max_/ cm^-1^: 1622, 3441;^1^H-NMR (400 MHz, DMSO-*d*_6_)*δ* 1.79 (s, 3H, CH_3_), 6.76 (s, 4H, 2NH_2_), 7.38-7.53 (m, 5H, Ar-H), 7.54 (s, 2H, 2CH); ^13^C-NMR (100 MHz, DMSO-*d*_6_)*δ* 159.3, 148.8, 148.1, 136.0, 134.3, 129.8,14.8; MS *m*/*z*(%): 394[M+, 2%]; Anal. calcd. for C_19_H_14_N_4_O_2_S_2_: C, 57.85; H, 3.58; N, 14.20; O, 8.11; S, 16.26; Found: C, 57.74; H, 3.52; N, 14.32; S, 16.34.

### 4,4'-(3-Methyl-4-phenylthieno[2,3-b]thiophene-2,5-diyl)dithiazol-2-amine (8b)

**8b** was prepared from thiourea following GP3 as a dark green powder crystals; Yield (75%); solid, mp 260–261°C; IR (KBr)ν_max_/ cm^-1^: 1620, 3439; ^1^H-NMR (400 MHz, DMSO-*d*_6_)*δ* 1.76 (s, 3H, CH_3_), 6.56 (s, 4H, 2NH_2_), 7.38-7.53 (m, 5H, Ar-H), 7.54 (s, 2H, 2CH); ^13^C-NMR (100 MHz, DMSO-*d*_6_)*δ* 160.1, 148.8, 148.1, 136.1, 134.3, 129.6, 128.8, 14.8; MS *m*/*z*(%): 426 [M+, 2%]; Anal. calcd. for C_19_H_14_N_4_S_4_: C, 53.49; H, 3.31; N, 13.13; S, 30.07 Found: C, 52.64; H, 3.51; N, 13.34; S, 30.32.

### 4,4'-(3-Methyl-4-phenylthieno[2,3-b]thiophene-2,5-diyl)bis(1H-imidazol-2-amine) (8c)

**8c** was prepared from guanidine following GP3 as a brown powder crystals; Yield (75%); solid, mp > 320°C; IR (KBr) ν_max_/ cm^-1^: 1624, 3420;^1^H-NMR (400 MHz, DMSO-*d*_6_)*δ* 1.87 (s, 3H, CH_3_), 6.74 (s, 4H, 2NH_2_), 7.38-7.53 (m, 5H, Ar-H), 7.51 (s, 2H, 2CH), 12.31 (s, 2H, 2NH); ^13^C-NMR (100 MHz, DMSO-*d*_6_)*δ* 158.4, 148.8, 148.1, 136.0, 134.3, 129.5, 128.8, 14.8; MS *m*/*z*(%): 392[M+, 2%]; Anal. calcd. for C_19_H_16_N_6_S_2_: C, 58.14; H, 4.11; N, 21.41; S, 16.34; Found: C, 57.64; H, 4.21; N, 21.31; S, 16.29.

### General procedure for the synthesis of compounds 9a,b (GP4)

A mixture of compound **2** (236 mg, 0.5 mmol) and aniline derivatives (2 equiv., 1 mmol) in EtOH (15 mL) was refluxed for 6–8 h in the presence of 0.5 mL of (TEA). The solid product was collected by filtration to give the corresponding products **9a**,**b**.

### 1,1'-(3-Methyl-4-phenylthieno[2,3-b]thiophene-2,5-diyl)bis(2-(phenylamino) ethanone) (9a)

**9a** was prepared from aniline following GP4 as a pale green powder crystals; Yield (90%); solid, mp > 320°C; IR (KBr) ν_max_/ cm^-1^: 1651, 3385; ^1^H-NMR (400 MHz, DMSO-*d*_6_)*δ* 2.02 (s, 3H, CH_3_), 4.42 (s, 4H, 2CH_2_), 6.22-7.57 (m, 15H, Ar-H), 7.61 (s, 2H, 2NH); ^13^C-NMR (100 MHz, DMSO-*d*_6_)*δ* 181.0, 147.5, 134.2, 129.7, 129.6, 129.1, 129.0, 120.0, 114.4, 113.9,67.9, 14.8; MS *m*/*z*(%): 496[M+, 3%]; Anal. calcd. for C_29_H_24_N_2_O_2_S_2_: C, 70.13; H, 4.87; N, 5.64; O, 6.44; S, 12.91; Found: C, 71.23; H, 4.37; N, 5.34; S, 12.96.

### 1,1'-(3-Methyl-4-phenylthieno[2,3-b]thiophene-2,5-diyl)bis(2-(4-chlorophenylamino) ethanone) (9b)

**9b** was prepared from *p*-chloroaniline following GP4 as a pale brown powder crystals; Yield (89%); solid, mp > 320°C:IR (KBr) ν_max_/ cm^-1^:1651, 3387;^1^H-NMR (400 MHz, DMSO-*d*_6_)*δ* 2.03 (s, 3H, CH_3_), 4.41 (s, 4H, 2CH_2_), 6.23-7.58 (m, 13H, Ar-H), 7.63 (s, 2H, 2NH); ^13^C-NMR (100 MHz, DMSO-*d*_6_)(ppm): 181.1, 147.5, 134.2, 129.7, 129.6, 129.1, 129.0, 120.0, 114.4, 113.9, 67.8, 14.8; *MS m*/*z*(%): 565[M+, 2%]; Anal. calcd. for C_29_H_22_C_l2_N_2_O_2_S_2_: C, 61.59; H, 3.92; N, 4.95; S, 11.34; Found: C, 60.79; H, 3.87; N, 4.90; S, 12.24.

### Biological activities

Various In vitro assays were performed to the assessment of biological activity of newly synthesized compounds. Results were presented here as means ± standard error from triplicate (n = 3) observation. IC_50_ values were calculated by using EZ-FIT, Enzyme kinetics software by Perrella Scientific.

### Anticancer activity

Cytotoxic activity of compounds was evaluated in 96-well flat-bottomed microplates by using the standard MTT (3-[4, 5-dimethylthiazole-2-yl]-2, 5-diphenyl-tetrazolium bromide, MP) colorimetric assay [39]. For this purpose, PC3 cells (Prostrate Cancer) were cultured in Dulbecco’s Modified Eagle Medium, supplemented with 10% of fetal bovine serum (FBS, PAA), 100 IU/mL of penicillin and 100 μ g/mL of streptomycin in 75 cm^2^ flasks, and kept in 5% CO_2_ incubator at 37°C. Exponentially growing cells were harvested, counted with haemocytometer and diluted with a particular medium with 5% FBS. Cell culture with the concentration of 1x10^5^ cells/mL was prepared and introduced (100 μL/well) into 96-well plates. After overnight incubation, medium was removed and 200 μL of fresh medium was added with different concentrations of compounds (1-30 μM). Stock solution, 20 mM of compounds were prepared in 100% DMSO and final concentration of DMSO at 30 μM is 0.15% .After 48 hrs, 200 μL MTT (0.5 mg/mL) was added to each well and incubated further for 4 hrs. Subsequently, 100 μL of DMSO was added to each well. The extent of MTT reduction to formazan within cells was calculated by measuring the absorbance at 570 nm, using a micro plate reader (Spectra Max plus, Molecular Devices, CA, USA). The cytotoxicity was recorded as concentration causing 50% growth inhibition (IC_50_) for PC3 cells. The percent inhibition was calculated by using the following formula:

% inhibition = 100-((mean of O.D of test compound – mean of O.D of negative control)/ (mean of O.D of positive control – mean of O.D of negative control)*100).

The results (% inhibition) were processed by using Soft- Max Pro software (Molecular Device, USA).

### *In vitro* antioxidant activity

Test samples were allowed to react with stable free radical, 1, 1-diphenyl-2-picrylhydrazyl radical (DPPH, *Wako Chemicals* USA, Inc.)) for half an hour at 37°C. Various concentrations of test samples (prepared in DMSO) were incubated with DPPH (300 μM; prepared in ethanol). After incubation, decrease in absorption was measured at 515 nm using a microplate reader (SpectraMax plus 384). Percentage radical scavenging activity (% RSA) by samples was determined, in comparison with a DMSO- treated control group.% Radical scavenging activity was calculated by using the formula given in statistical analysis section [40].

### *In vitro β*-glucuronidase inhibition assay

*β*-Glucuronidase inhibitory activity was determined by the spectrophotometric method by measuring the absorbance at 405 nm of *p*-nitrophenol formed from the substrate (*p*-nitrophenyl-*β*-D-glucuronide N1627-250 mg (Sigma Aldrich). The total reaction volume was 250 μL. The compound (5 μL) was dissolved in DMSO (100%), which becomes 2% in the ultimate assay (250 μL) and the similar conditions were used for standard (D-saccharic acid 1, 4-lactone, Sigma Aldrich). The reaction mixture contained 185 μL of 0.1 M acetate buffer, 5 μL of test compound solution, 10 μL of (1U) enzyme solution (G7396-25KU, Sigma Aldrich) was incubated at 37°C for 30 min. The plates were read on a multiplate reader (SpectraMax plus 384) at 405 nm after the addition of 50 μL of 0.4 mM *p*-nitrophenyl-*β*-D-glucuronide. All assays were performed in triplicate. IC_50_ Values were calculated by using EZ-Fit software (Perrella Scientific Inc., Amherst, MA, U.S.A.). These values are the mean of three independent readings [41].

### *In vitro α*-glucosidase inhibition assay

*α*-Glucosidase inhibition assay was performed spectrophotometrically. *α*-Glucosidase from Saccharomyces cerevisiae (G0660-750UN, Sigma Aldrich), was dissolved in phosphate buffer (pH 6.8., 50 mM). Test compounds were dissolved in 70% DMSO. In 96-well plates, 20 μL of test sample, 20 μL of enzyme and 135 μL of buffer were added and incubated for 15 minutes at 37°C. After incubation, 25 μL of *p*-nitrophenyl- *α* -D-glucopyranoside (0.7 mM, Sigma Aldrich) was added and change in absorbance was monitored for 30 minutes at 400 nm. Test compound was replaced by DMSO (7.5% final) as control. Acarbose (Acarbose, Sigma Aldrich) was used as a standard inhibitor [42].

## Competing interests

The authors declare that they have no competing interests.

## Authors’ contributions

YNM proposed the subject, designed the study, helped in the results and discussion. SA carried out the synthesis of all the products. AB and AMA conceived the study and participated in its design, results and discussion, and coordination. SY and CMI carried out the biological assay. AB and SY prepared draft the manuscript. All the authors read and approved the final manuscript.

## Supplementary Material

Additional file 1 **Supporting information.** Selected copies of spectrum (^1^H-NMR, ^13^C-NMR, IR, and MS) for some synthesized compounds. Click here for file
