# Supplementary material for: Synthesis, reactions and biological activity of some new bis-heterocyclic ring compounds containing sulphur atom
Source: Chem Cent J. 2013 Jul 8;7:112. doi: 10.1186/1752-153X-7-112 (PMC3728144; doi:10.1186/1752-153X-7-112)
Supplement: Additional file 1 — Supporting information. Selected copies of spectrum (1H-NMR, 13C-NMR, IR, and MS) for some synthesized compounds. [file 1752-153X-7-112-S1.pdf]

SUPPORTING INFORMATION

- **Selected copies of spectrum (^{1}H-NMR, ^{13}C-NMR, IR, and MS) for some synthesized compounds.**

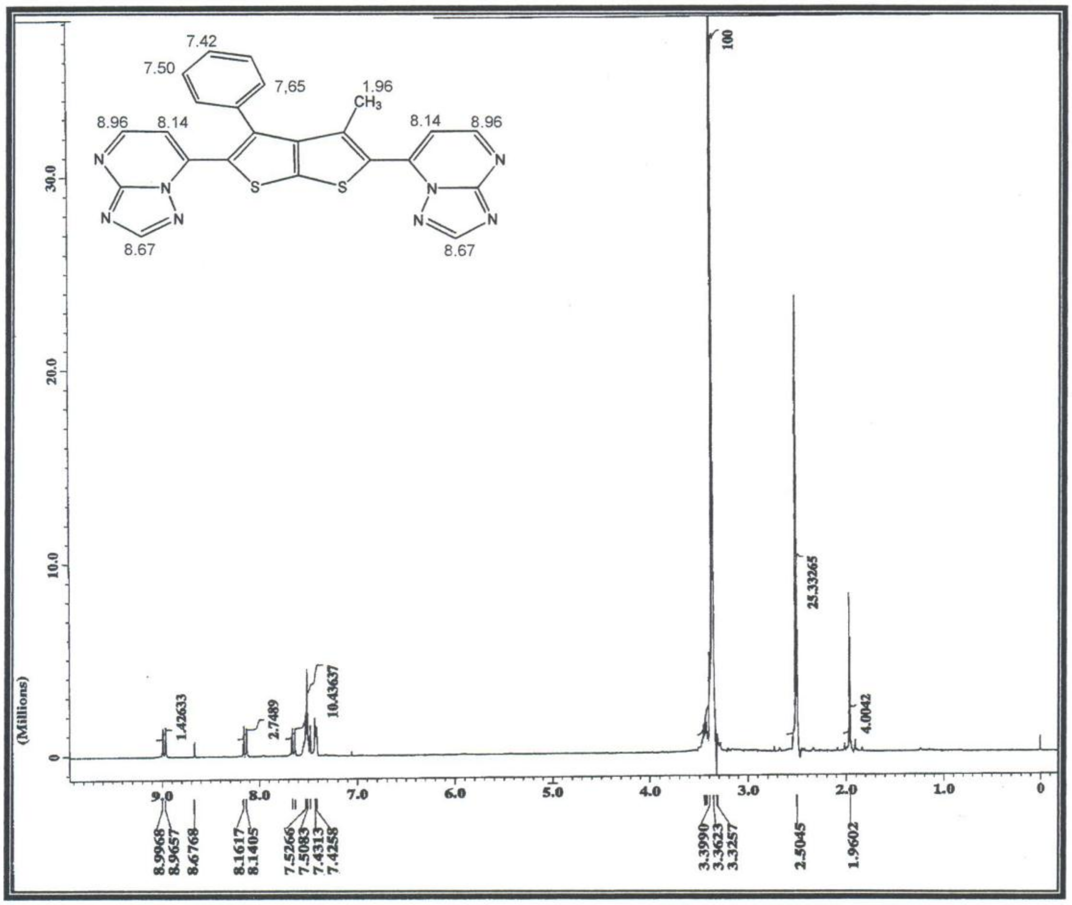

Figure S1. ^{1}H-NMR (400 MHz, DMSO-d_6) of **6.**

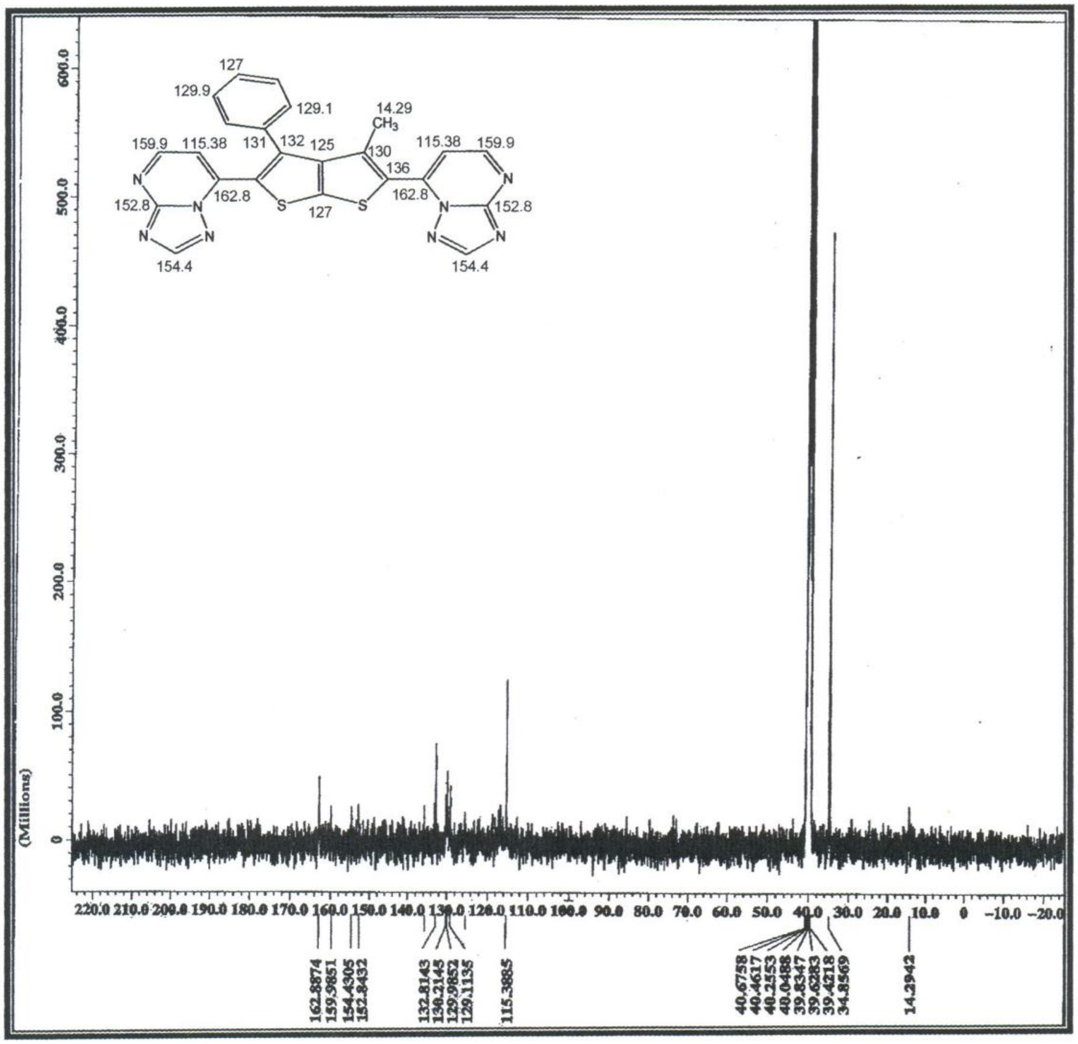

Figure S1. ^{13}C-NMR spectra (400 MHz, DMSO-d_6) of **6.**

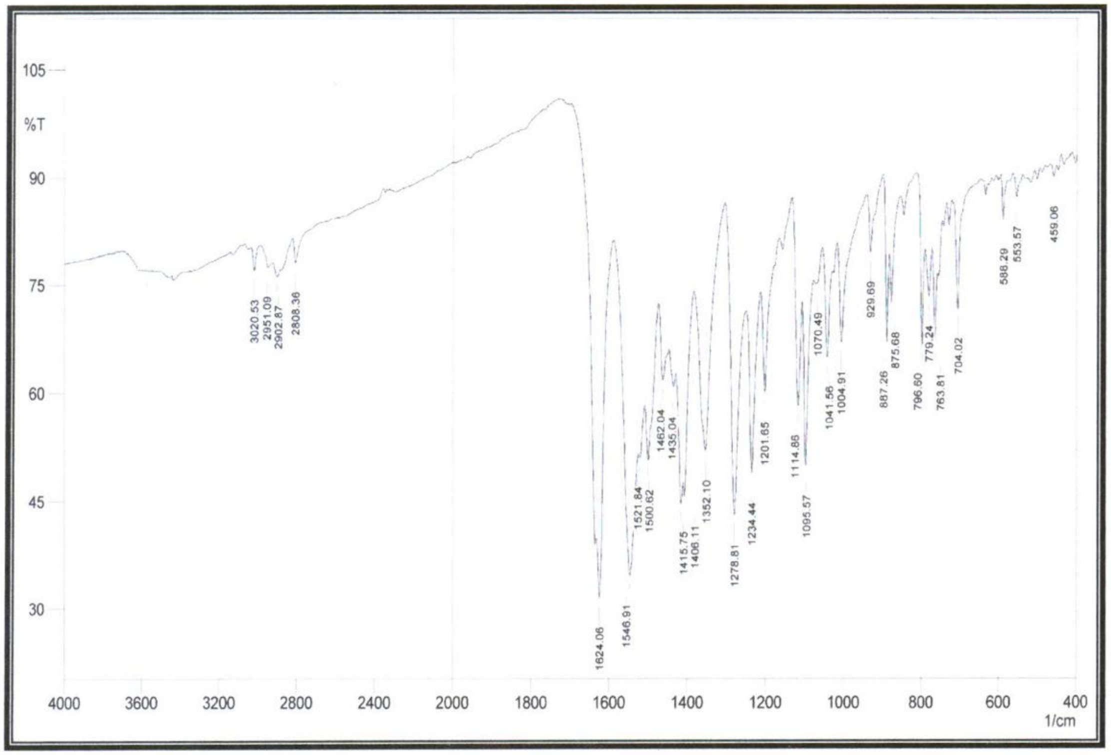

Figure S1. IR spectra (KBr) of **6.**

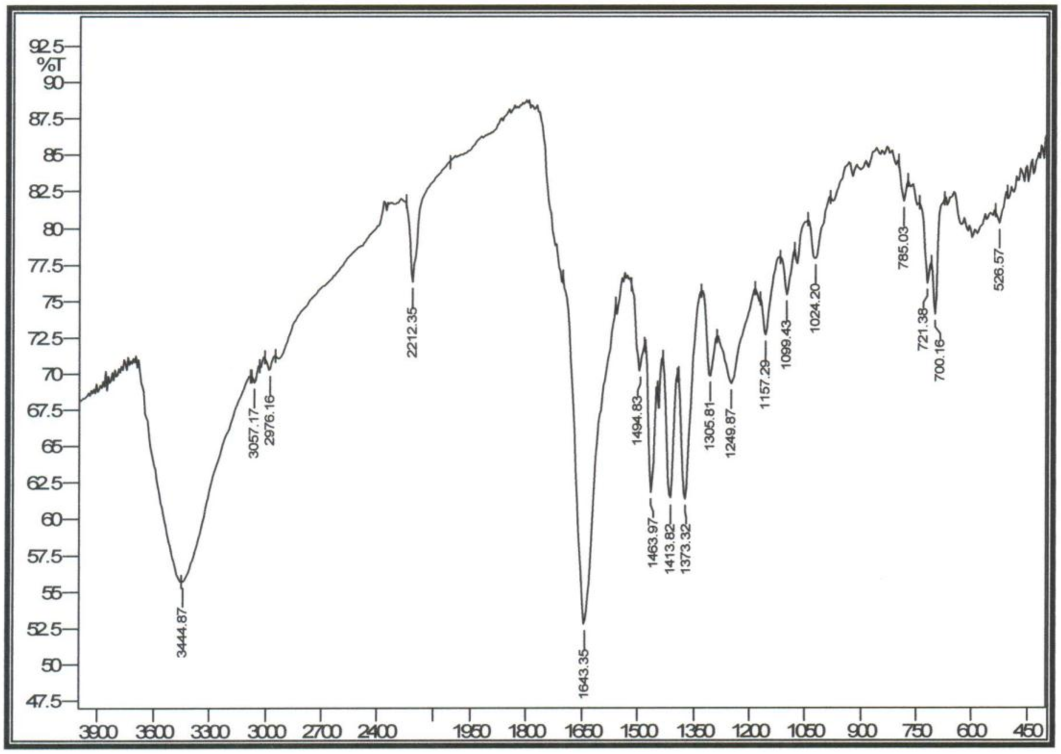

Figure S2. IR spectra (KBr) of **5.**

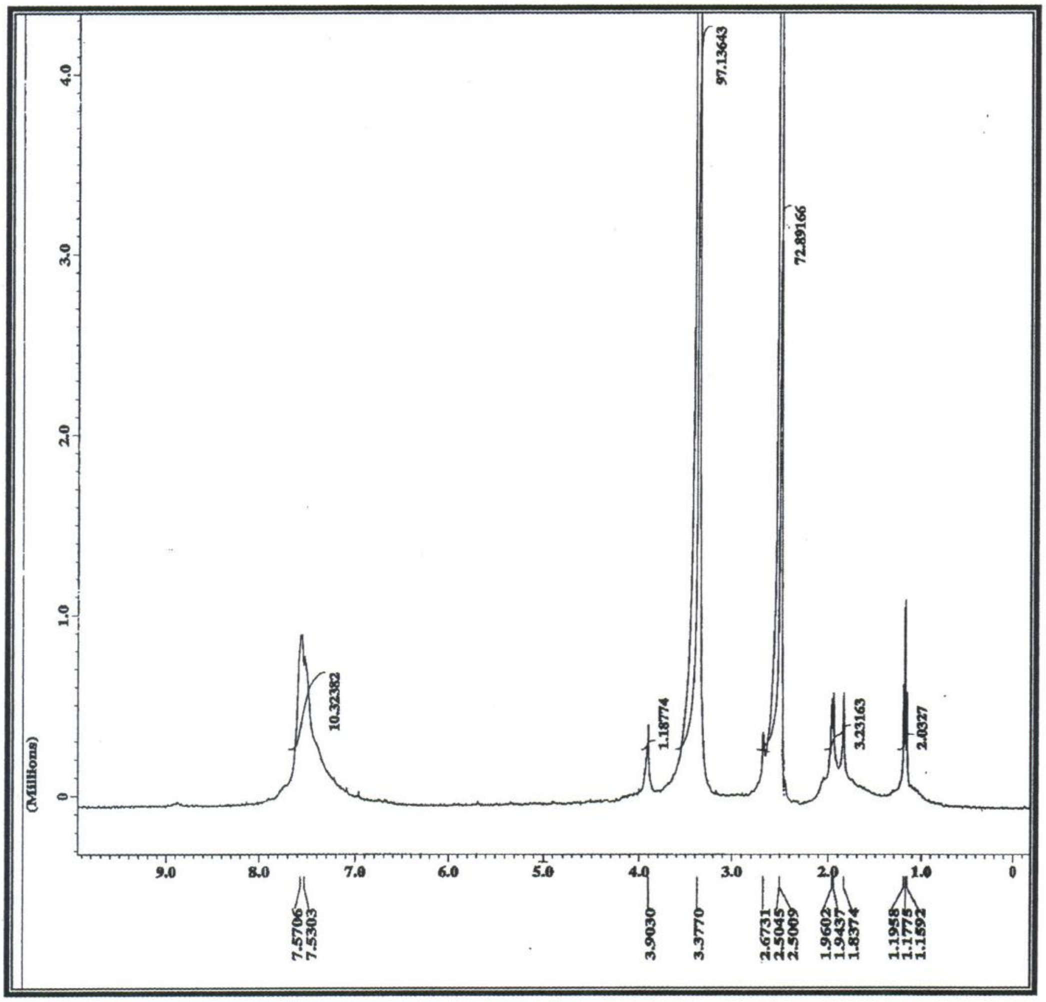

Figure S2. ^{1}H-NMR (400 MHz, DMSO-d_6) of **5.**

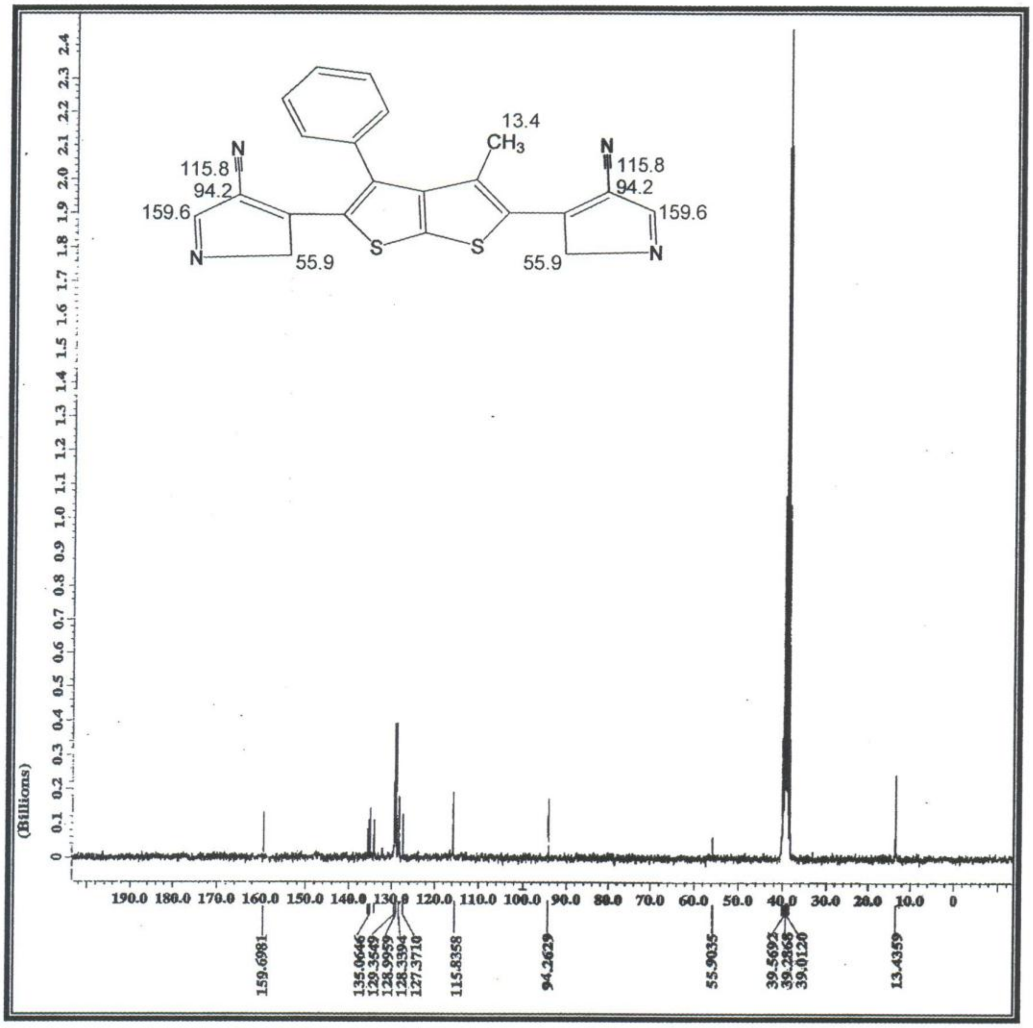

Figure S2. ^{13}C-NMR spectra (400 MHz, DMSO-d_6) of **5.**

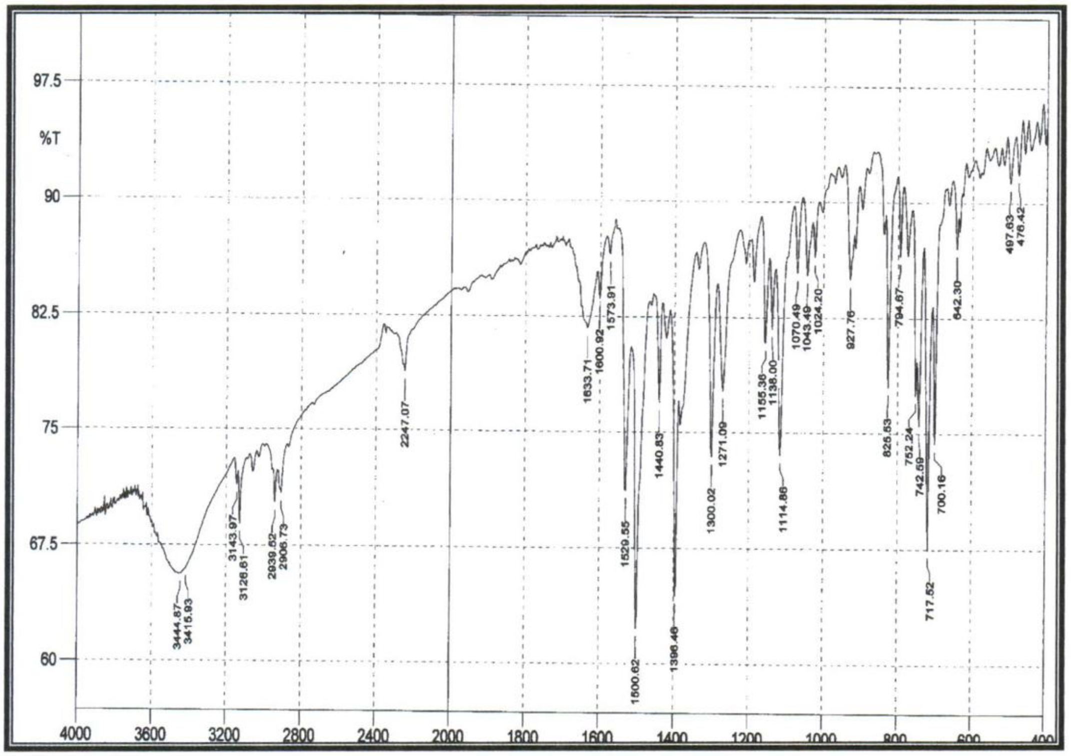

Figure S3. IR spectra (KBr) of **3.**

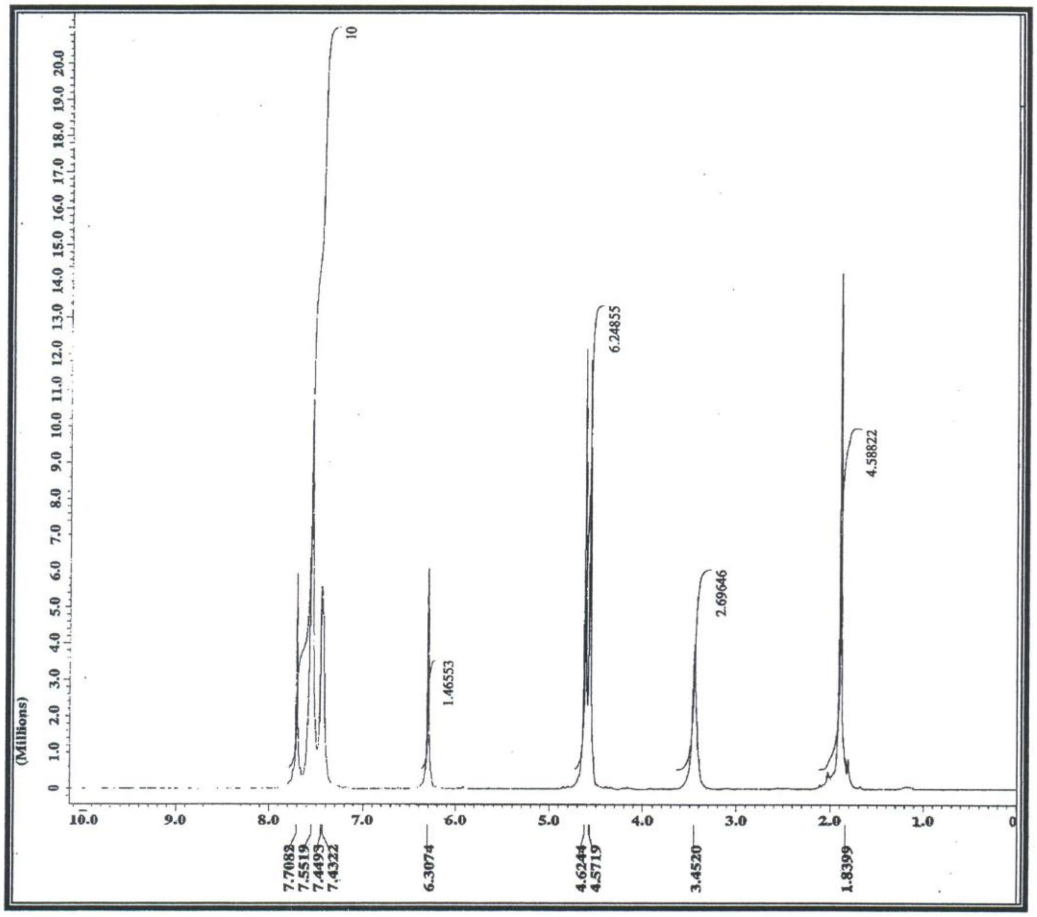

Figure S3. ^{1}H-NMR (400 MHz, DMSO-d_6) of **3**

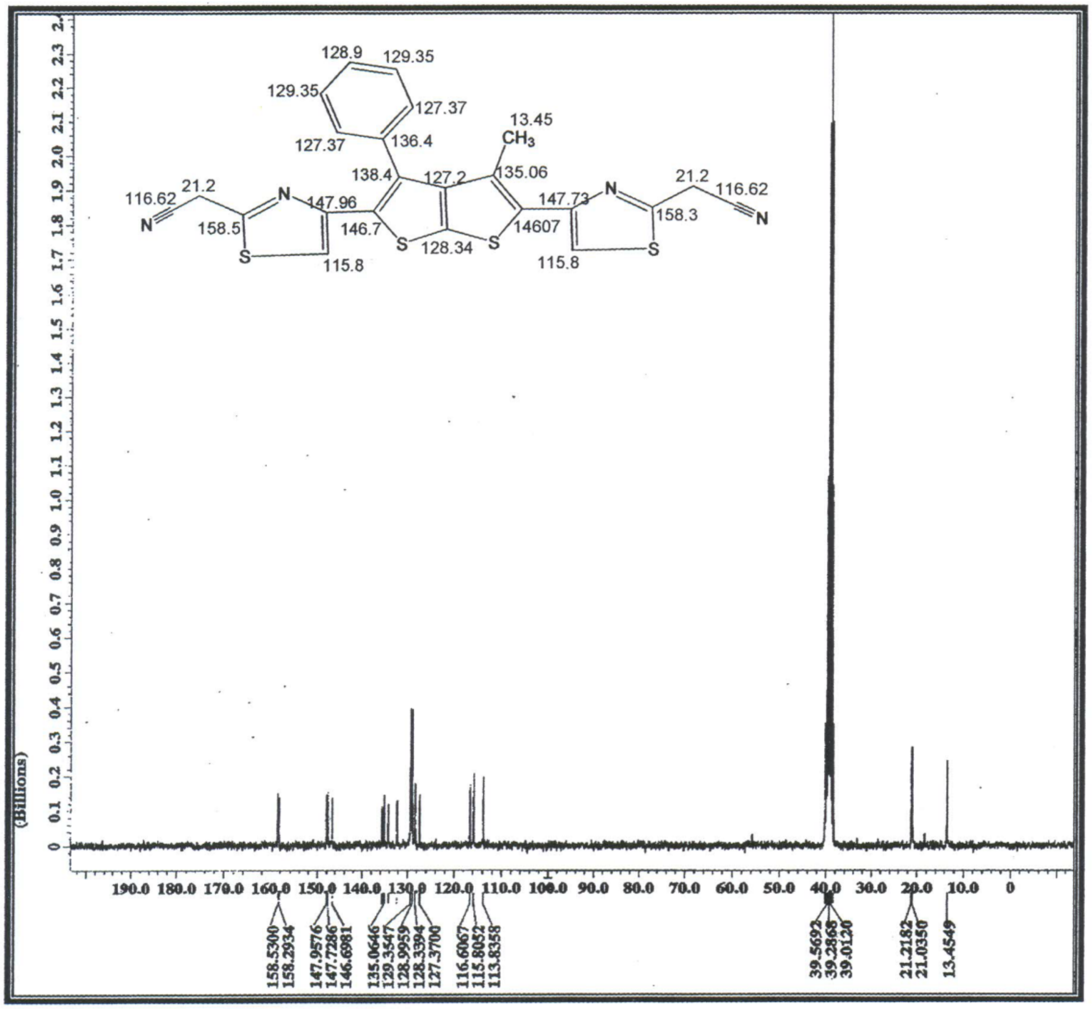

Figure S3. ^{13}C-NMR spectra (400 MHz, DMSO-d_6) of **3**

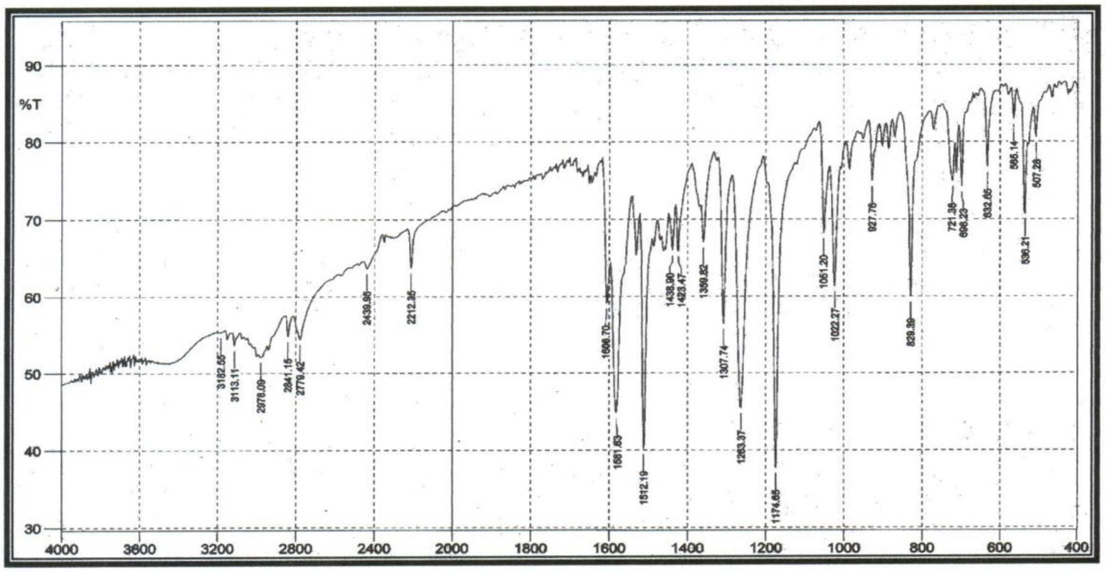

Figure S4. IR spectra (KBr) of **4a.**

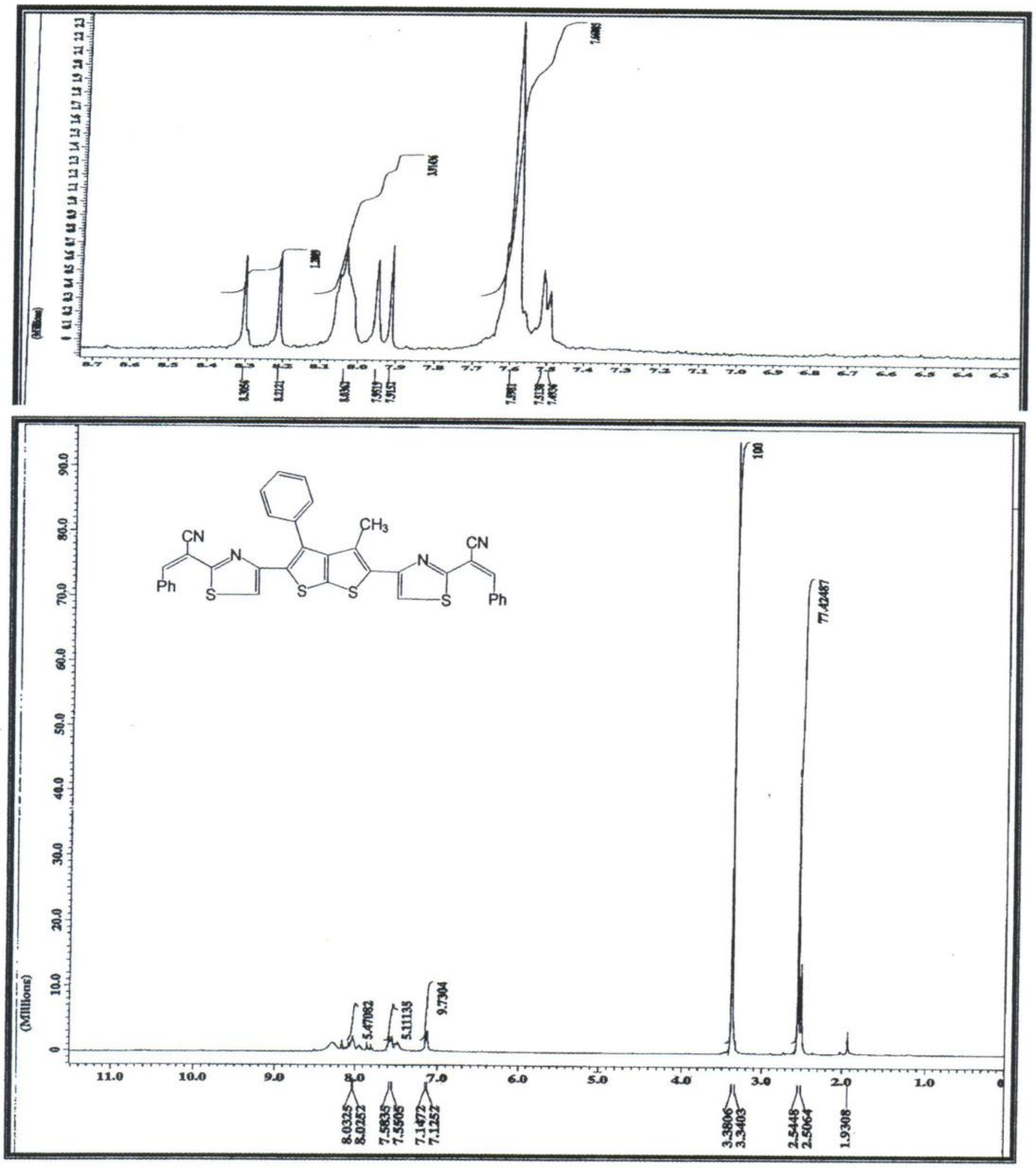

Figure S4. ^{1}H-NMR (400 MHz, DMSO-d_6) of **4a.**

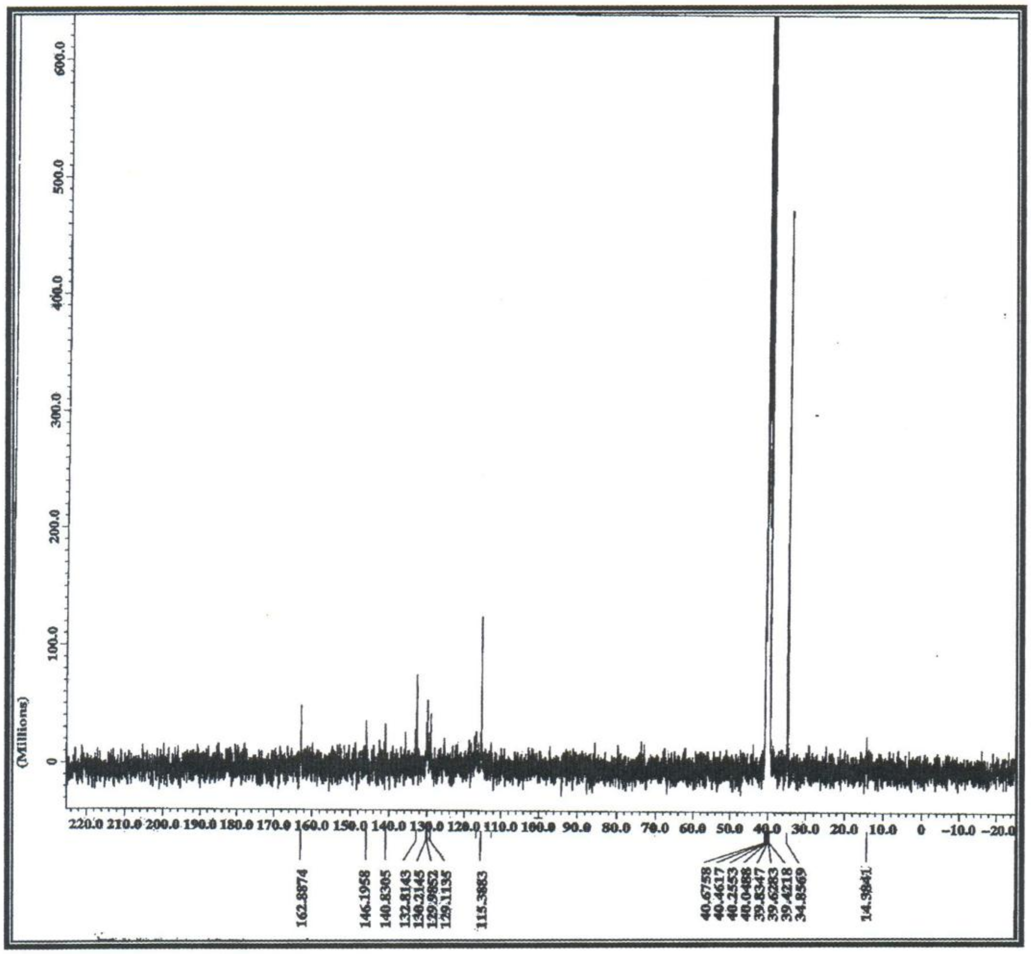

Figure S4. ^{13}C-NMR spectra (400 MHz, DMSO-d_6) of **4a.**

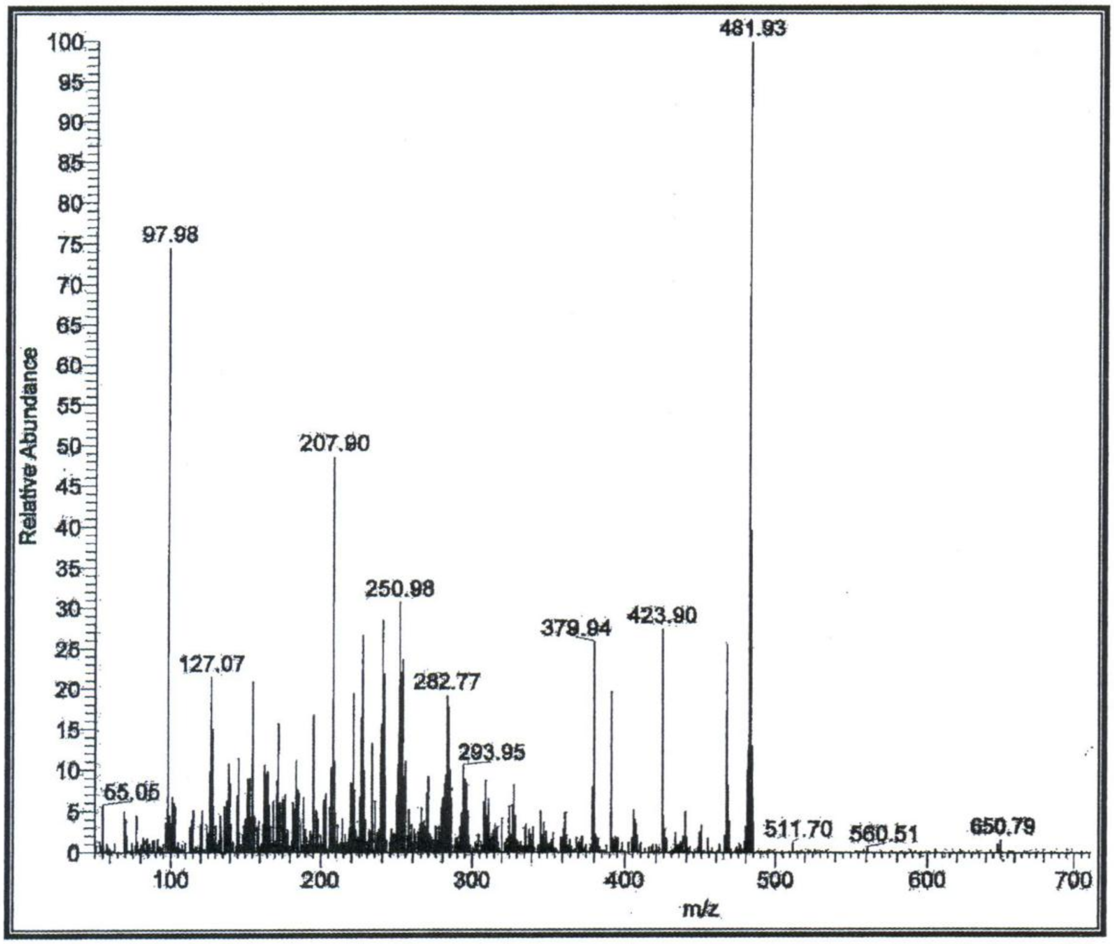

Figure S4. MS of **4a**.

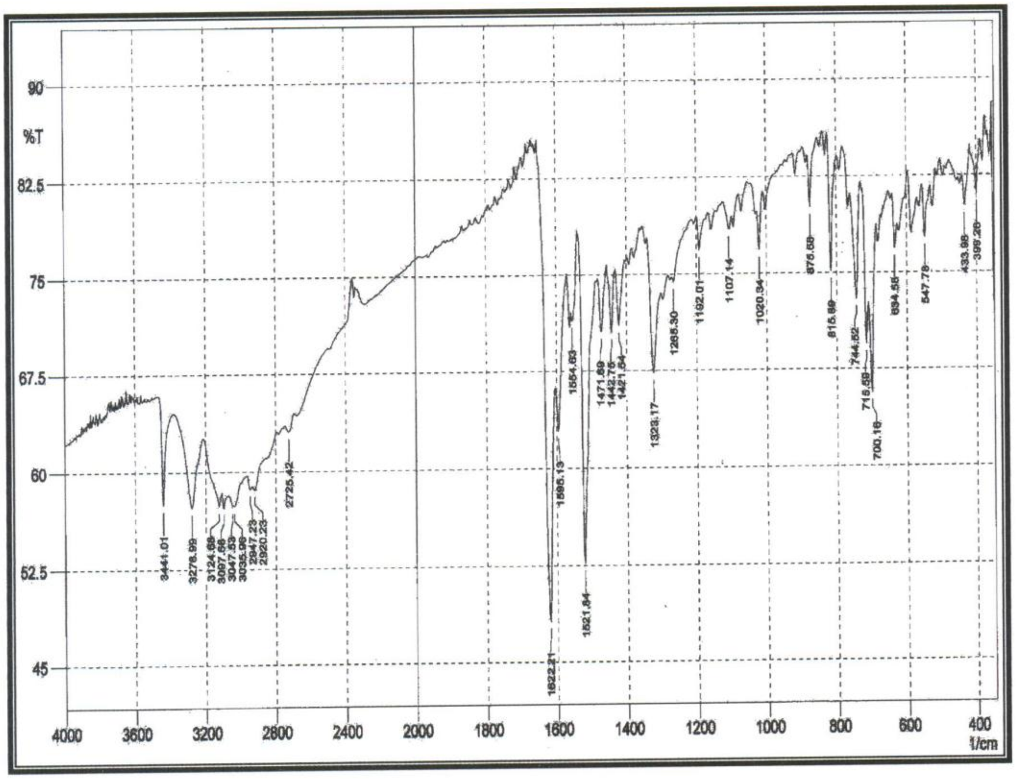

Figure S5. IR spectra (KBr) of **8a**.

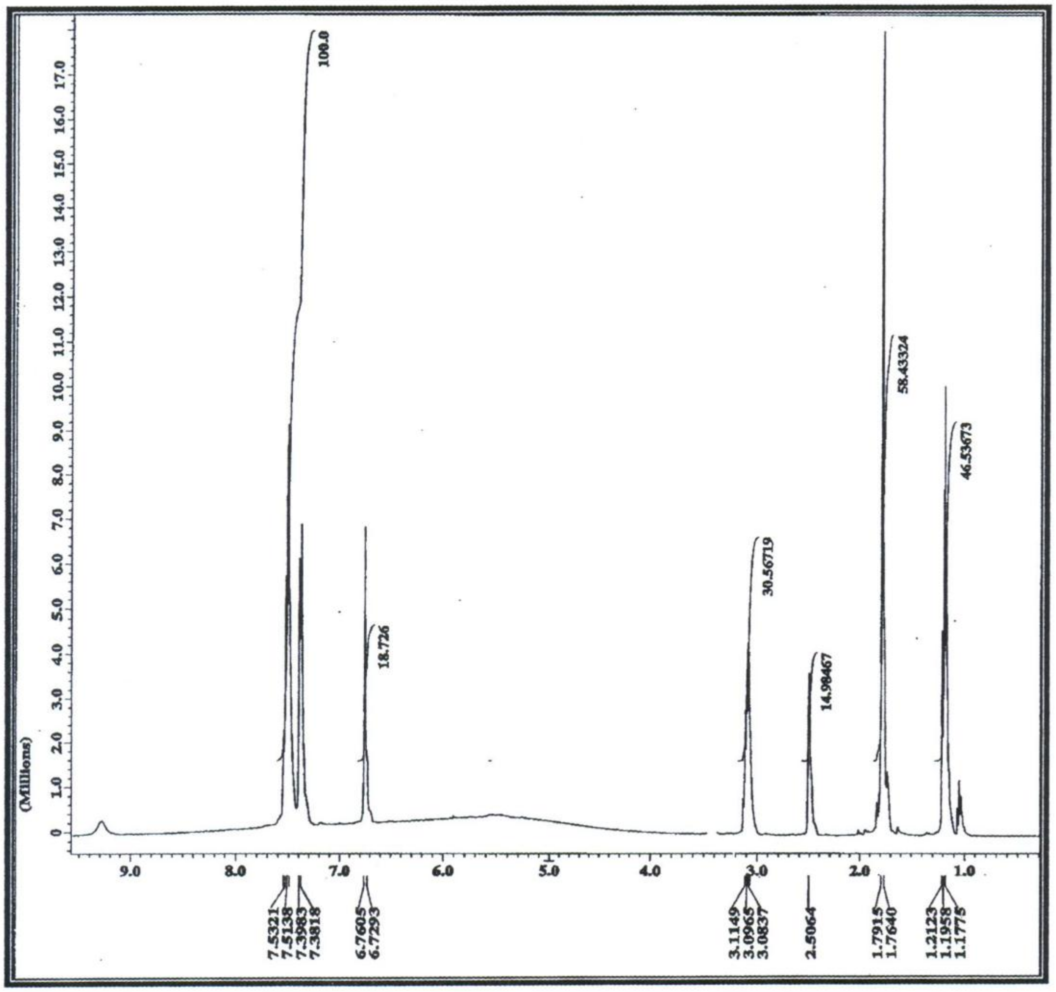

Figure S5. ^{1}H-NMR spectra (400 MHz, DMSO-d_6) of **8a.**

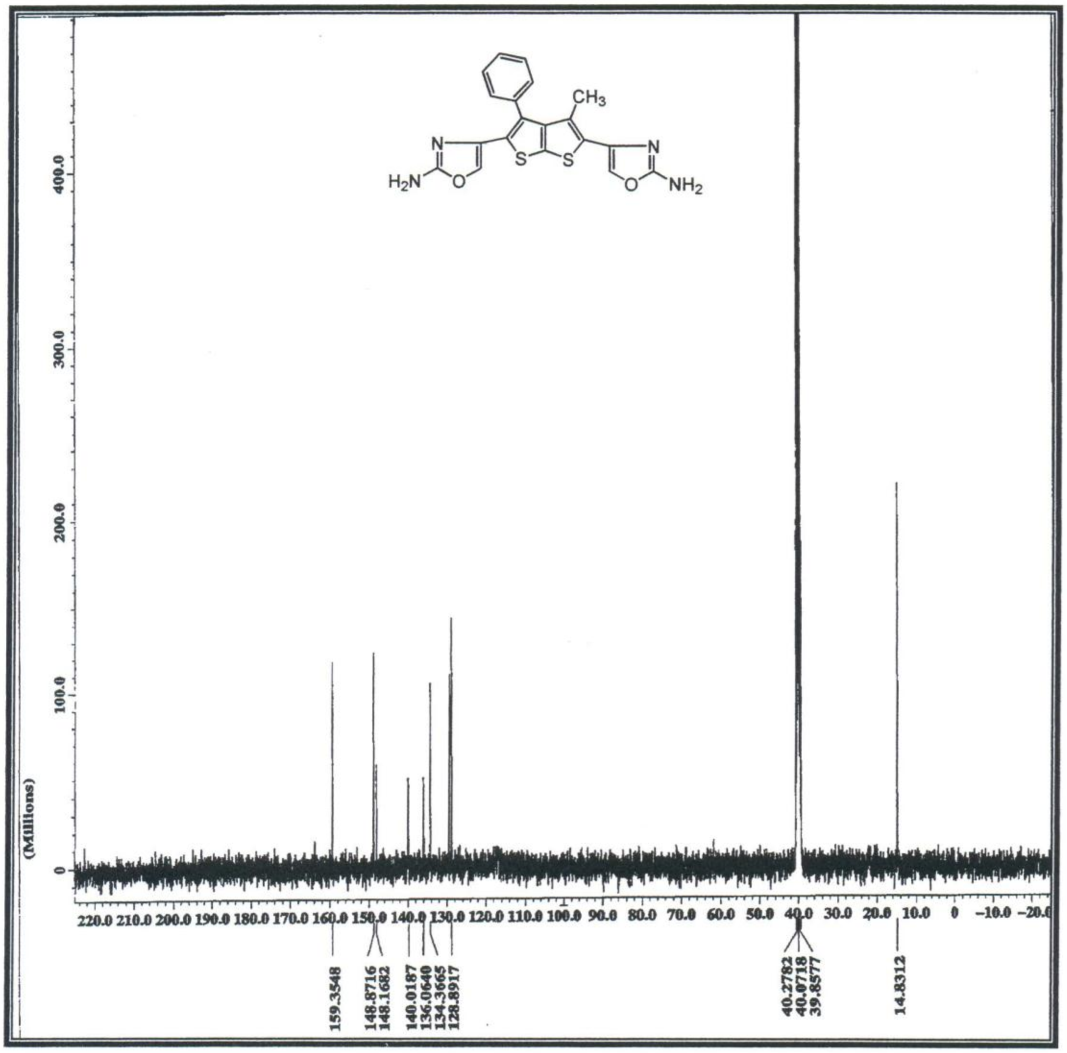

Figure S5. ^{13}C-NMR spectra (400 MHz, DMSO-d_6) of **8a.**

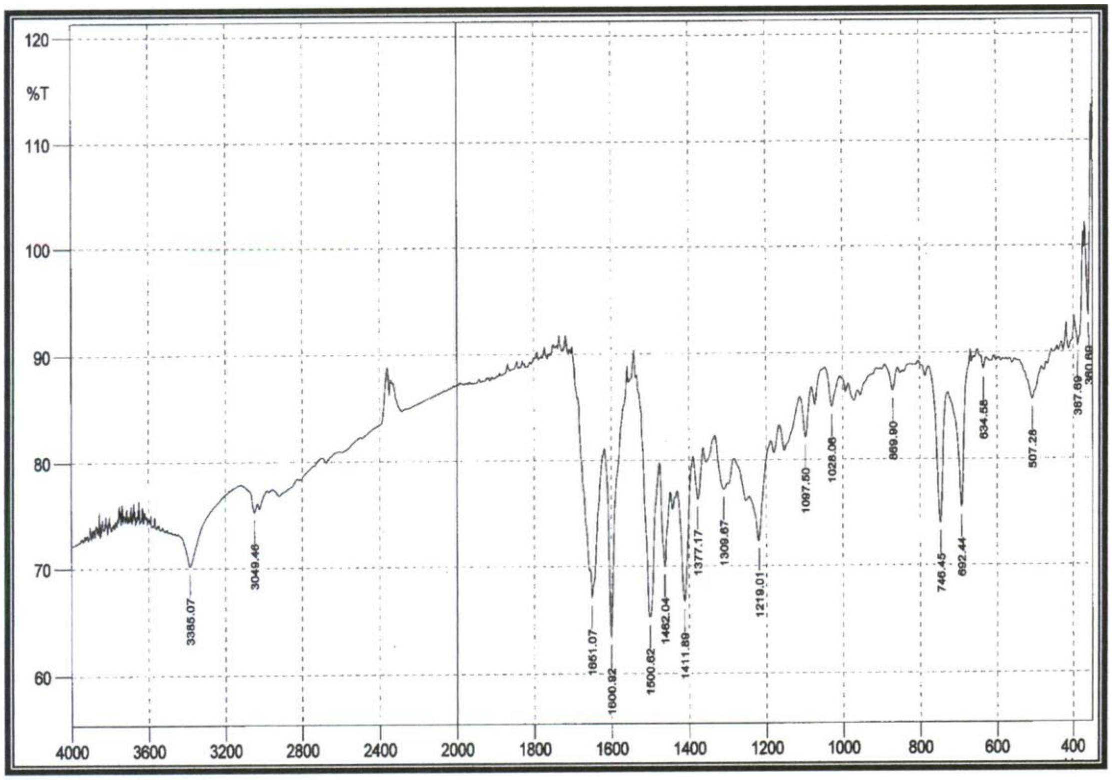

Figure S6. IR spectra (KBr) of **9a**.

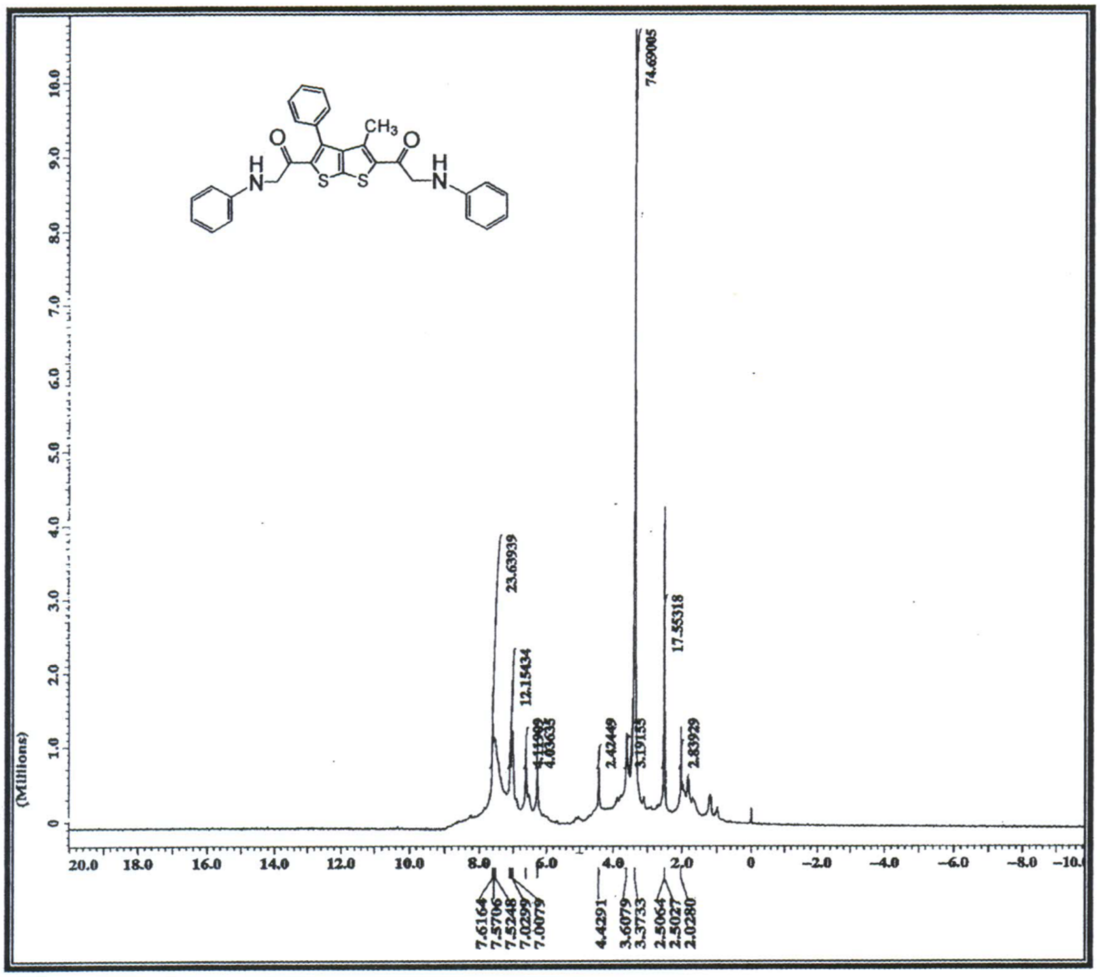

Figure S6. ^{1}H-NMR spectra (400 MHz, DMSO-d_6) of **9a.**
